# Biochemical characterization and ^1^H NMR based metabolomics revealed *Melicope lunu-ankenda* leaf extract a potent anti-diabetic agent in rats

**DOI:** 10.1186/s12906-017-1849-2

**Published:** 2017-07-10

**Authors:** Mizher Hezam AL-Zuaidy, Muhammad Waseem Mumtaz, Azizah Abdul Hamid, Amin Ismail, Suhaila Mohamed, Ahmad Faizal Abdul Razis

**Affiliations:** 1 Department of Food Science, Faculty of Food Science and Technology, Universiti Putra Malaysia, 43400 Serdang, Selangor Malaysia; 2 Faculty of Medicine and Health Sciences, Universiti Putra Malaysia, 43400 Serdang, Selangor Malaysia; 3 Institute of Bioscience, Universiti Putra Malaysia, 43400 Serdang, Selangor Malaysia; 4 Ministry of Iraqi Trade, State Company for Grain Processing, Baghdad, Iraq; 5 Department of Chemistry, University of Gujrat, Gujrat, Punjab 50700 Pakistan; 6 Institute of Tropical Agriculture and Food Security, Universiti Putra Malaysia, 43400 Serdang, Selangor Malaysia

**Keywords:** Type 2 diabetes, *Melicope lunu-ankenda* extract, Anti-diabetic, ^1^H Nmr, Metabolomics

## Abstract

**Background:**

Type 2 diabetes mellitus (T2DM) is a metabolic disorder characterized by continuous hyperglycemia associated with insulin resistance and /or reduced insulin secretion. There is an emerging trend regarding the use of medicinal plants for the treatment of diabetes mellitus. *Melicope lunu-ankenda* (ML) is one of the *Melicope* species belonging to the family Rutaceae. In traditional medicines, its leaves and flowers are known to exhibit prodigious health benefits. The present study aimed at investigating anti-diabetic effect of *Melicope lunu-ankenda* (ML) leaves extract.

**Methods:**

In this study, anti-diabetic effect of ML extract is investigated in vivo to evaluate the biochemical changes, potential serum biomarkers and alterations in metabolic pathways pertaining to the treatment of HFD/STZ induced diabetic rats with ML extract using ^1^H NMR based metabolomics approach. Type 2 diabetic rats were treated with different doses (200 and 400 mg/kg BW) of *Melicope lunu-ankenda* leaf extract for 8 weeks, and serum samples were examined for clinical biochemistry. The metabolomics study of serum was also carried out using ^1^H NMR spectroscopy in combination with multivariate data analysis to explore differentiating serum metabolites and altered metabolic pathways.

**Results:**

The ML leaf extract (400 mg/kg BW) treatment significantly increased insulin level and insulin sensitivity of obese diabetic rats, with concomitant decrease in glucose level and insulin resistance. Significant reduction in total triglyceride, cholesterol and low density lipoprotein was also observed after treatment. Interestingly, there was a significant increase in high density lipoprotein of the treated rats. A decrease in renal injury markers and activities of liver enzymes was also observed. Moreover, metabolomics studies clearly demonstrated that, ML extract significantly ameliorated the disturbance in glucose metabolism, tricarboxylic acid cycle, lipid metabolism, and amino acid metabolism.

**Conclusion:**

ML leaf extract exhibits potent antidiabetic properties, hence could be a useful and affordable alternative option for the management of T2DM.

## Background

Type 2 diabetes mellitus (T2DM) is a metabolic disorder characterized by continuous hyperglycemia associated with insulin resistance and /or reduced insulin secretion [1]. The pathophysiology of T2DM has not been completely understood, and there are views, that link T2DM with insulin signaling deficiencies, obesity and reactive oxygen species (ROS). Obesity is an excessive fat build up in the body and is one of the major risk factor in the etiology of T2DM, and a positive relationship between insulin resistance and obesity has also been reported [2].

A number of animal models have been examined to evaluate pathogenesis of diabetes and its associated complications. Rats fed a HFD following the administration of low dose of streptozotocin (STZ) develop T2DM in almost similar way to that of human’s type 2 diabetes [3–5]. Metabolomics is an emerging tool for comprehensive description of endogenous metabolites representing the “metabolome”. It helps in building a profile of small molecules and provides snapshot of whole metabolic pathways in plants, animals or human. It is being considered as one of the most widely employed analytical tool for the investigation of diabetes mellitus, due to its ability to elaborate biochemical pathways and their interactive roles in systemic metabolism [6, 7]. Metabolomics studies are normally based on nuclear magnetic resonance (NMR) spectrometry in combination with multivariate data analysis [8, 9].

Treatment of diabetes using synthetic drugs such as biguanides, in particular, metformin and sulfonylureas is often accompanied with adverse side effects and normally fails to correct the major biochemical disorders and diabetic complications. Alternatively, herbal extracts and phytopharmaceuticals have now been recognized as emphatic therapeutic options in treating T2DM with minimal side effects. Medicinal plants can be used either as an anti-diabetic remedy alone or in combination with other hypoglycemic drugs or insulin [10, 11].

*Melicope lunu-ankenda* (ML) is one of the *Melicope* species belonging to the family Rutaceae and is ubiquitously found all around the world, particularly, in Asia and Australia. In Malaysia twenty-four *Melicope* species have been identified [12]. The leaves are normally consumed as salad and condiment for food flavoring. The leaves and flower are also traditionally being consumed to manage hypertension, menstrual disorder and fever etc. [13, 14]. Presence of secondary metabolites including; alkaloids, flavonoids, acetophenones and coumarins in several *Melicope* species further demonstrate their health benefits [15, 16].

Previous work in the same laboratory ascertained the potent anti-diabetic activity of ML in vitro [17]. In the present study, the anti-diabetic activity of the ML extract is further explored in vivo using high fat diet-STZ induced diabetic rats. Moreover, NMR based metabolomics approach was employed to evaluate potential serum biomarkers and changes in metabolic pathways involved in the treatment of obese diabetic rats with ML extract.

## Methods

### Plant materials and extraction

Mature leaves of ML were collected from Agriculture University Park, UPM. A voucher specimen (SK2507/14) was deposited at the herbarium, Institute of Bioscience, University Putra Malaysia, and the specie was identified as *Melicope lunu-ankenda.* The leaves were washed, dried with tissue paper and were immediately quenched with liquid nitrogen followed by freeze-drying. The dried leaves were then ground to powder. The leaf powder was then kept in sealed plastic bags and stored at −80 °C until further analysis.

Ten grams of powdered leaves were dissolved in a solvent system consisted of ethanol-water (60:40). The mixture was then vortexed and ultrasonicated for 1 h., at room temperature, centrifuged (13,000 rpm for 10 min) and filtered with filter paper (whatman No:1). Evaporation of the solvent was conducted using rotary evaporator at 40 °C until a viscous extract was obtained. The crude extract was then freeze-dried until constant weight to ensure removal of the residual water (72 h) and kept at −80 °C until further analysis. The percentage yield of extract was 20.8 ± 1.9%.

### Experimental animals and design

The experiments were carried out with 60 male Sprague Dawley rats (aged 6 weeks, with body weights of 204.5 g on the average) purchased from the Animals Resource Unit, Faculty of Veterinary Medicine, UPM. A study approval was obtained from Institutional Animal Care and Use Committee, University Putra Malaysia (UPM\IACUC\AUP-R002\2015). The rats were housed at a temperature (25 ± 5 °C) in 12 h light and dark cycles, with 65 ± 5% humidity, free access to standard diet and water for a week prior to the treatment. After acclimatization, rats were randomly divided into two groups based on the assigned diets: First group, normal group (NG) (*n* = 12), was fed normal diet (ND), which was standard diet (Specialty Feeds Pty. Ltd., Australia) included 4.8% fat, 59.4% carbohydrate, 4.8% crude fiber and 20% protein with a metabolic energy level of 3343 kcal/kg. While the second group (*n* = 48) was fed high fat diet (HFD) (Altromin, C1090–45) included 22% fat, 43% carbohydrates, 21% protein and 3% fiber and provided overall 4630 kcal/kg, comprising of 43% of calories from fat, 38% of calories from carbohydrates and 18% of calories from protein.

The body weights of the rats were measured on weekly basis. After 8 weeks, % body weight gain of the rats was measured using the following formula:

$$ \mathrm{The}\ \mathrm{percentage}\ \mathrm{weight}\ \mathrm{gain}=\frac{\mathrm{Body}\ \mathrm{weight}\ \mathrm{on}\ \mathrm{specific}\ \mathrm{week}\ \left(\mathrm{g}\right)\hbox{--} \mathrm{Baseline}\ \mathrm{body}\ \mathrm{weight}\ \left(\mathrm{g}\right)}{\mathrm{Baseline}\ \mathrm{body}\ \mathrm{weight}}\times 100 $$

**Fig. 1:**
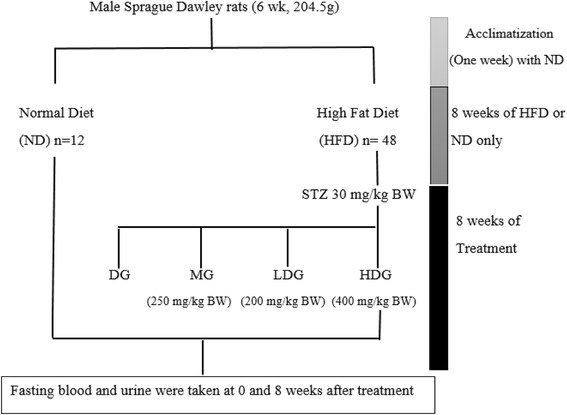
Schematic diagram of the experimental design to evaluate the antidiabetic effect of 60% ethanolic of ML leaf extract in obese diabetic male Sprague-Dawley rats

Type 2 diabetes was induced as per pervious method [18] with slight modifications. The rats in second group fed with HFD, after an overnight fasting were intraperitoneally injected with a low dose of STZ (30 mg per kg BW, mixed with 0.1 M cold citrate buffer, pH 4.5). After 3 days of STZ injection, fasting blood glucose (from tail vein) was measured using glucometer (Roche, Mannheim, Germany). Rats with fasting blood glucose level (≥16.6 mmol/L) were considered to be diabetic [4]. In addition to the normal group (NG), the diabetic rats were randomly divided further into four groups (each group consisted of 12 rats) including diabetic group (DG), diabetic group treated with metformin (MG), diabetic group treated with low dose ML extract (LDG) and diabetic group treated with high dose ML extract (HDG) as described below (Fig. 1).

**Normal group (NG):** Negative control rats fed normal diet and received 0.03% (*w*/*v*) of carboxymethyl cellulose (CMC) for 8 weeks.

**Diabetic Group (DG):** HFD-STZ induced diabetic rats received 0.03% (*w*/*v*) CMC for 8 weeks.

**Diabetic Group treated with metformin (MG):** HFD-STZ induced diabetic rats treated with metformin (250 mg/kg BW) suspended in 0.03% (*w*/*v*) CMC for 8 weeks.

**Diabetic Group treated with low dose ML extract (LDG):** HFD-STZ induced diabetic rats treated with low dose of ML ethanolic leaf extract (200 mg/kg BW) suspended in 0.03% (*w*/*v*) CMC for 8 weeks.

**Diabetic Group treated with high dose ML extract (HDG):** HFD-STZ induced diabetic rats treated with high dose of ML ethanolic leaf extract (400 mg/kg BW) suspended in 0.03% (*w*/*v*) CMC for 8 weeks.

### Food and water consumption measurements

Three rats were housed in one cage containing 90 g of food and 500 mL of water. Unconsumed food and remaining water were weighed/measured after 24 h. These measurements were conducted daily.

### Sacrifice of rats

After 8 weeks of treatment, the rats were weighed, blood glucose levels measured and sacrificed by cardiac puncture under anesthesia (ketamine and xylazine) after an overnight fasting. The blood was centrifuged for 15 min at 1500 g, serum was collected and was kept at −80 °C until further analysis. Rat’s organs (liver, kidney and spleen) were also collected and weighed. All the rats were handled according to “the international principles of the use and handling of experimental animals (United States National Institute of Health, 1985)”.

### Biochemical analyses

Serum of the rats at week 0 and week 8 of treatment was investigated for its total cholesterol (TC), triglycerides (TG), low-density lipoprotein (LDL) and high-density lipoprotein (HDL) in vitro using Roche/ Hitachi cobas C system (Roche Diagnostic GmbH, Sandhofer Strasse Mannheim). Serum was also analyzed for alkaline phosphatase (ALP), aspartate transaminase (AST) and alanine aminotransferase (ALT) as markers for hepatobiliary disease. Tests were conducted on a Roche/ Hitachi cobas C system. The kidney function tests were performed using kinetic colorimetric assay in order to measure serum creatinine on a Roche/ Hitachi cobas C system along with quantitative measurement of urea/urea nitrogen in serum.

### Determination of insulin

Serum insulin of the rats at week 0 and week 8 of treatment was measured using enzyme-linked immune absorbent assay (ELISA) kit (Mercodia Rat Insulin ELISA, Uppsala Sweden) following manufacturer’s instructions.

### Determination of glucose level

Before and after stipulated time period of the treatment, fasting blood from the tail vein of the rates was collected and blood was subjected to blood glucose level measurement using a glucometer (Roche, Mannheim, Germany).

### Calculation of insulin resistance and insulin sensitivity

Insulin resistant and insulin sensitivity were measured using the method reported by [19].

Insulin resistant was measured by Homeostasis Model (HOMA-IR) as follows:

$$ \mathrm{HOMA}\hbox{-} \mathrm{IR}=\frac{\mathrm{Fasting}\ \mathrm{insulin}\ \mathrm{levels}\ \left(\mathrm{ug}/\mathrm{L}\right)\ \mathrm{x}\ \mathrm{fasting}\ \mathrm{glucose}\ \mathrm{levels}\ \left(\mathrm{mmol}/\mathrm{L}\right)}{22.5} $$

Insulin sensitivity was calculated using the following formula:

$$ \mathrm{Insulin}\ \mathrm{sensitivity}=\frac{\mathrm{Fasting}\ \mathrm{serum}\ \mathrm{insulin}\ \mathrm{level}\ \left(\mathrm{ug}/\mathrm{L}\right)}{\mathrm{Fasting}\ \mathrm{serum}\ \mathrm{glucose}\ \mathrm{level}\ \left(\mathrm{mmol}/\mathrm{L}\right)} $$

### ^1^H NMR analysis of serum

^**1**^ H NMR analysis of the serum samples was carried out as per method described by [20]. The thawed serum samples were centrifuged at 12000×g for 10 min. Two hundred *µ*L of the supernatant was mixed with 400 *µ*L of phosphate buffer (containing 0.2% TSP) and was transferred into NMR tubes (5 mm). Water suppressed Carr-Purcell Meiboom-Gill (CPMG) spin-echo pulse was carried out along with NOESY-preset experiments to suppress the broad signals due to macromolecules. The CPMG spectra were then recorded with 128 transients, with an acquisition time (1.36 s), relaxation delay (2.0 s), and number of loops (*n* = 80). Moreover, two-dimensional ^1^H-^1^H J resolved and ^1^H- ^13^C HMBC analysis were also performed to further confirm the identity of the metabolites.

### ^1^ H NMR spectral data reduction and multivariate data analysis

Chenomx NMR Suite (Chenomx, Calgary, Canada) was used for the characterization of metabolites. Non zero filled spectra were manually phased, the baseline was corrected, calibrated to TSP at 0.00 ppm, and using the profiler module, the 0–10 ppm processed spectra were segmented (0.04 ppm). Residual water (*δ* 4.75–*δ* 4.85) and urea signals (*δ* 5.50–*δ* 6.00 ppm) were excluded from the analysis. Remaining bins were then normalized to sum of the spectral integrals, extracted with Microsoft Excel, and for multivariate data analysis imported into Simca-P software (Umetrics, Umea, Sweden). Multivariate data analysis (PCA, OPLS-DA etc) was then performed for biomarkers analysis among the lean groups and obese diabetic groups to elaborate any metabolite changes associated with ML treatment.

### Statistical analysis

Experimental data was analyzed utilizing MINITAB version 16. Statistical differences between samples and controls were evaluated by one-way ANOVA test followed by Tukey’s test and paired T Test. Results are expressed as mean ± standard deviation (SD). A difference in the mean values with *p* < 0.05 was considered to be statistically significant.

## Results

### Induction of obesity

**Fig. 2:**
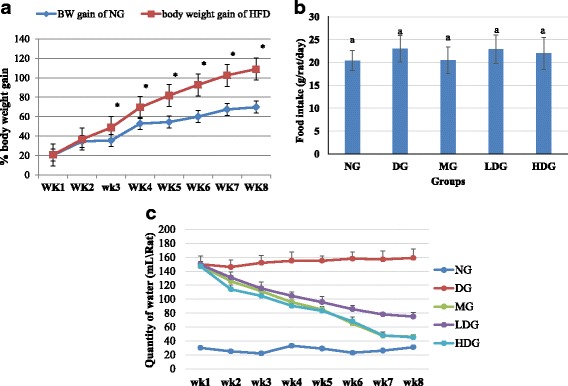
**a** Body weight gain of normal diet group (NG) group and high fat diet (HFD) groups after 8 weeks of dietary manipulation, **b** Average of food intake **c** Average of drinking water consumption from baseline to the end of treatment by normal diet group (NG), diabetic group (DG), metformin group (MG) low dose group (LDG) and high dose group (HDG) Values with* and different letters indicate significance difference (*p* < 0.05)between difference groups as shown by the analysis of variance (ANOVA) using MINITAB VERSION 16

After 8 weeks of dietary manipulation, the Sprague Dawley rats fed a HFD showed significant weight gain (*p* < 0.05) as compared to the rats fed with ND (Fig. 2a). When the body weight gain of the NG was compared with that of HFD rats, starting from week 3, a significant difference was observed in body weights of the rats by 35.5 and 48.7%, respectively. An increase in body weight gain was revealed as intervention progress. At week 8 significant differences (*p* < 0.05) in the body weight gain of both groups were depicted. The percent weight gain of rats fed with ND and HFD was 69.7 ± 8.8% and 108.9 ± 12.3%, respectively. The results ascertained that, the use of HFD diet successfully induced obesity in all male Sprague Dawley rats after an administration period of 8 weeks. After induction of obesity, the rats on HFD were ingested with STZ to induce diabetes.

### Effect of ML extract on the food and water consumption

The food consumption during the period of treatment was same for all groups of rats (*p* > 0.05) (Fig. 2b), whereas the water consumption was decreased significantly (*p* < 0.05) during the treatment period after initial administration with ML extract (200 and 400 mg/ kg BW) and metformin (250 mg/ kg BW), which reached closer to normal group (NG) at the end of experiment (Fig. 2c). The obese diabetic group (DG) consumed significantly (*p* < 0.05) higher quantity of water comparative to other groups.

### Effect of ML extract on the body weight changes

**Fig. 3:**
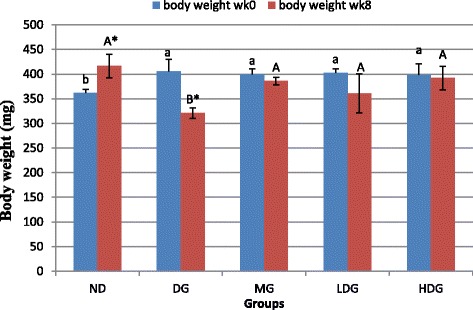
Body weight of normal diet group (NG), diabetic group (DG), metformin group (MG) low dose group (LDG) and high dose group (HDG) before and after treatment

The effect of ML extract on the body weights of rats is described in Fig. 3. Obese diabetic rats demonstrated significant (*p* < 0.05) reduction in body weights as compared to the healthy rats. The weight of obese diabetic rats continued to decrease till the end of experiment. However, the rats treated with low dose (200 mg/kg BW) of ML extract, high dose (400 mg/kg BW) of ML extract and metformin (250 mg/kg BW) prevented the body weight loss (381.5 ± 19.2, 392.0 ± 24.3 and 385.8 ± 8.0 g, respectively) and there was non-significant difference (*p* > 0.05) comparative to the healthy rats (416.5 ± 24.0 g).

### Effect of ML extract on fasting serum insulin

**Fig. 4:**
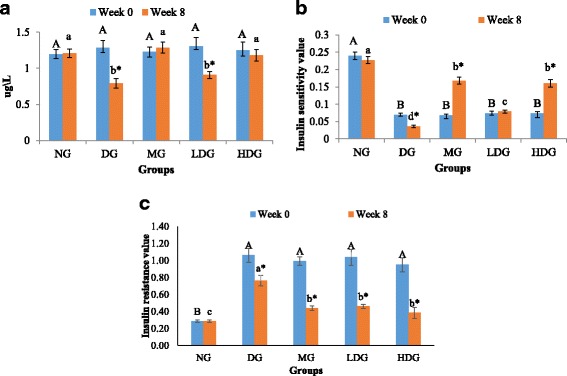
**a** Serum insulin levels, **b** Insulin sensitivity values, **c** Insulin resistance values of normal diet group (NG), diabetic group (DG), metformin group (MG) low dose group (LDG) and high dose group (HDG) before and after treatment

Effect of oral administration of the ML leaf extract on fasting serum insulin level is shown in Fig. 4a. At the week 0 of treatment after HFD/STZ induction, there was no significant (*p* > 0.05) difference between all the obese diabetic groups and healthy group regarding their insulin level. After 8 weeks of treatment, a significant (*p* < 0.05) decrease (34.5%) in the insulin level of obese diabetic group was observed comparative to the healthy rats group. However, with low dose ML extract treatment, the decrease in the insulin level was 25.1% comparative to the healthy group. On the other hand, the results revealed that, there was a pronounced effect of high dose ML extract treatment on the insulin levels, and the results were also comparable with those of metformin treatment (Fig. 4a). This implies that, the treatment with high dose of ML extracts maintained the insulin level almost equivalent to that of healthy rats.

### Effect of ML extract on insulin sensitivity and insulin resistance

The effect of ML extract on the insulin sensitivity is presented in Fig. 4b. It was observed that, the HFD/STZ induced diabetic groups showed significant (*p* < 0.05) reduction in the levels of insulin sensitivity compared with those of healthy rats group before treatment. Treatment with high dose of ML extract considerably improved insulin sensitivity. It was depicted that the treatment with 400 mg/kg BW ML extract and 250 mg/kg BW metformin significantly (*p* < 0.05) enhanced insulin sensitivity compared with that of untreated obese diabetic group by 34.4% and 36.6%, respectively at the completion of experimental period.

Figure 4c shows effect of ML extract on insulin resistance. On the completion of experimental period, all of the treated obese diabetic groups i.e., treated with 200 mg/kg BW of ML extract, 400 mg/kg BW of ML extracts and 250 mg/kg BW of metformin showed a significant (*p* < 0.05) drop in the insulin resistance compared to the untreated obese diabetic group by 38.9%, 50.0% and 49.1%, respectively.

### Effects of ML extract on the serum lipid profile

**Fig. 5:**
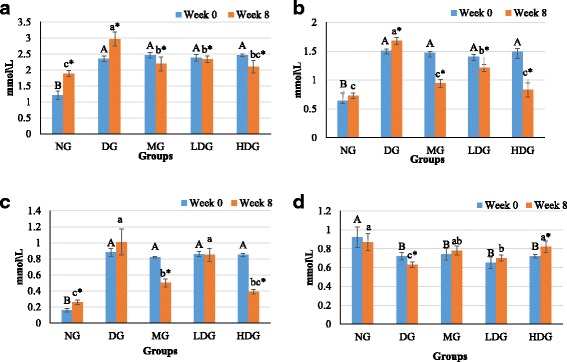
**a** Total cholesterol levels, **b** Triglycerides levels, **c** LDL levels and **d** HDL levels of normal diet group (NG), diabetic group (DG), metformin group (MG) low dose group (LDG) and high dose group (HDG) before and after treatment

Experimental rats fed with HFD following injection of STZ exhibited higher levels of total cholesterol (TC) and triglyceride (TG) (2 fold) as compared to those of healthy rats (Fig. 5a & b). Administration of both the low dose (200 mg/kg BW) and high dose (400 mg/kg BW) of ML extract for 8 weeks showed significant (*p* < 0.05) reduction in both TC and TG levels. High dose of ML extract reduced the TC (2.07 ± 0.22 mmol/L) and TG (0.85 ± 0.14 mmol/L) levels near or equal to healthy rat’s TC (1.9 ± 0.05 mmol/L) and TG (0.74 ± 0.067) levels, respectively.

Low density lipoprotein (LDL) levels in the serum of all obese diabetic groups were significantly (*p* < 0.05) increased when compared with that of normal group before treatment (Fig. 5c). After 8 weeks of treatment, with high dose of ML extract and metformin significant (*p* < 0.05) decrease in LDL levels was observed with values 0.39 ± 0.04 mmol/L and 0.5 ± 0.06 mmol/L, respectively as compared with that of untreated obese diabetic group (0.94 ± 0.08 mmol/L), and non-significant (*p* > 0.05) difference was depicted between those treated with high dose of ML extract and normal group (0.26 ± 0.04 mmol/L).

On the other hand, significant (*p* < 0.05) increase in the HDL levels (0.84 ± 0.04 mmol/L) was observed in the obese diabetic rats treated with high dose of ML extract (Fig. 5d) comparative to HDL levels (0.54 ± 0.03 mmol/L) of obese diabetic group. The results regarding HDL levels of high dose ML extract treated group were comparable with those of metformin treated group with non-significant (*p* > 0.05) difference. Moreover, the elevated HDL levels were close to those of healthy group (0.86 ± 0.11 mmol/L).

### Effect of ML extracts on liver enzymes activities

Rats fed with HFD for 8 weeks followed by STZ induction showed significant (*P* < 0.05) rise in the levels of liver enzymes ALP and ALT compared with those of rats on normal diet, but with no effect on AST enzyme (Table 1). The treatment with 400 mg/kg BW of ML extract and metformin significantly (*P* < 0.05) decreased the ALP enzyme levels i.e., 76.4 ± 6.3 U/L and 70.8 ± 6.1 U/L, respectively as compared with that of untreated obese diabetic group (165.0 ± 13.9 U/L) after 8 weeks of treatment. On the other hand, when compared with the liver enzyme activities of healthy rats group, the AST and ALT levels were depicted to be 82.4 ± 2.8 U/L and 34.3 ± 3.0 U/L, respectively and there was non-significant (*P* > 0.05) differences in serum AST and ALT levels after 8 week of treatment with high dose of ML extract (84.3 ± 2.4 U/L and 39.3 ± 3.7 U/L) and metformin (85.6 ± 5.2 U/L and 37.4 ± 3.1 U/L), respectively.

**Table 1:** The body weights, serum alanine aminotransferase (ALT), alkaline phosphatase (ALP) aspartate aminotransferase (AST), creatinine and urea levels before and after treatment

	Before treatment					After treatment				
	NG	DG	MG	LDG	HDG	NG	DG	MG	LDG	HDG
ALT(U/L)	37.0 ± 2.0^b^	54.4 ± 2.9^a^	52.8 ± 3.1^a^	51.5 ± 3.6^a^	55.1 ± 3.9 ^a^	34.3 ± 3.0^c^	64.5 ± 4.6^a*^	37.4 ± 3.1^c*^	55.2 ± 5.0^b^	39.3 ± 3.7^c*^
ALP (U/L)	61.0 ± 6.8^b^	159.3 ± 9.0^a^	160.8 ± 9.2^a^	161.00 ± 6.9^a^	158.5 ± 4.5^a^	63.6 ± 6.7^c^	165.0 ± 13.9^a^	70.8 ± 6.1^c*^	111.2 ± 8.7^b*^	76.4 ± 6.3^c*^
AST(U/L)	79.0 ± 3.3^a^	90.1 ± 6.7^a^	89.7 ± 5.9^a^	88.7 ± 5.3^a^	89.8 ± 6.3^a^	82.4 ± 2.8^c^	101.4 ± 10.2^a*^	85.6 ± 5.2^bc^	92.6 ± 6.5^b^	84.3 ± 2.4^bc^
Creatinine (umol/L)	53.5 ± 3.8^b^	61.3 ± 3.2^a^	57.3 ± 2.3^ab^	59.3 ± 3.6^ab^	58.3 ± 4.2^ab^	62.3 ± 7.3^b^	70.8 ± 7.1^a*^	64.8 ± 4.1^ab^	66.5 ± 4.0^ab^	62.5 ± 6.0^ab^
Urea (mmol/L)	6.4 ± 0.2^a^	6.7 ± 0.2^a^	6.9 ± 0.7^a^	6.7 ± 0.9^a^	6.8 ± 0.8^a^	6.1 ± 0.93^b^	12.5 ± 2.0^a*^	6.6 ± 0.4^b^	10.6 ± 0.9^a*^	7.0 ± 0.9^b^

*ND* Normal diet, *DG* diabetic group, *MG* metformin group, *LDG* low dose group, *HDG* high dose group. Significance difference (*p* < 0.05) among different groups within same period of treatment is shown by different letters, whereas * shows significance (*p* < 0.05) difference within same group at different period of treatment

### Effects on the serum urea and Creatinine levels

The effect of ML extract on serum urea levels is shown in Table 1. In comparison with the healthy rats group, serum urea and creatinine values were significantly (*p* < 0.05) higher in the obese diabetic group. The treatment with ML extract (400 mg/kg BW) and metformin showed significant reduction in serum urea levels compared with those of obese diabetic group. The treatment with 400 mg/kg BW of ML extracts and 250 mg/kg BW of metformin decreased the serum urea levels by 43.0% and 45.2%, respectively as compared to the obese diabetic group, and there was non-significant (*P* > 0.05) differences between them comparative to the healthy group. On the other hand, non-significant (*p* > 0.05) differences were observed in the creatinine levels between the rats treated with 200 mg/kg BW of ML extracts, 400 mg/kg BW of ML extracts and metformin compared with those of healthy group at the week 8 of treatment.

### Effect of ML extract on body organs weight

The liver weights of obese diabetic rats showed significant increase (*p* < 0.05) compared to those of healthy rats (Table 2). However, the treatment with 200 mg/kg BW, 400 mg/kg BW of ML extract and metformin (250 mg/kg BW) for 8 weeks resulted in significant decrease of the liver weights comparative to the obese diabetic rats group. Although, there were no significant differences (*p* > 0.05) at the week8 of treatment between 400 mg/kg BW of ML extract and metformin treated obese diabetic groups compared with that of healthy group. The liver weights of obese diabetic rats decreased by 17.47% and 17.75% after treatment with 400 mg/kg BW of ML extract and 250 mg/kg BW of metformin, respectively when compared with untreated obese diabetic rats group. Significant (*p* < 0.05) increase in kidney weight by 41.7% was also observed for obese diabetic rats as compared to the healthy rats (Table 2). However, 200 mg/kg BW and 400 mg/kg BW of ML extracts and metformin treated obese diabetic rats showed significant reduction in their kidney weights compared to those of the untreated obese diabetic rats at week8 of administration. At the end of experimental period there was non-significant (*p* > 0.05) difference between 400 mg/kg BW of ML extract and metformin treated obese diabetic rats regarding their kidney weights. In addition, spleen weight was significantly (*p* < 0.05) deceased in untreated obese diabetic rats group compared with healthy rats group (Table 2) and non-significant (*p* > 0.05) difference was observed between 400 mg/kg ML extracts treated obese diabetic rats group with healthy group based on their spleen weights.

**Table 2:** Weight of rat body organs liver, kidney and spleen

Weight of organs (g)	NG	DG	MG	LDG	HDG
Liver	10.22 ± 1.02^c^	14.25 ± 1.18^a^	11.72 ± 1.29^bc^	12.97 ± 0.96^ab^	11.76 ± 0.83^bc^
Kidney	2.47 ± 0.23^c^	3.50 ± 0.35^a^	2.75 ± 0.26^bc^	3.22 ± 0.30^ab^	2.90 ± 0.45^abc^
Spleen	0.71 ± 0.07^a^	0.52 ± 0.09^b^	0.46 ± 0.08^b^	0.52 ± 0.05^b^	0.59 ± 0.10^ab^

*NG* Normal diet, *DG* diabetic group, *MG* metformin group, *LDG* low dose group, *HDG* high dose group. Significance difference (*p* < 0.05) among different groups is shown by different letters

### ^1^H NMR spectra of serum metabolites before (week 0) treatment

**Fig. 6:**
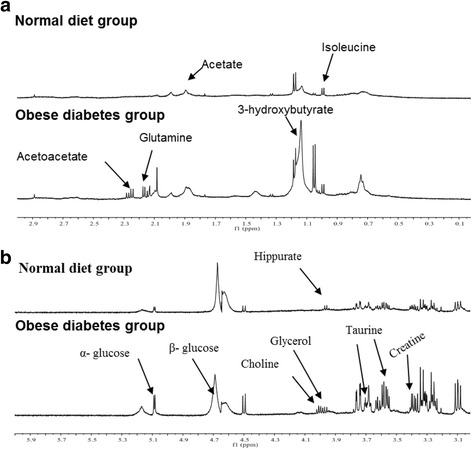
Typical 500 MHz ^1^HNMR serum **a** (0 to 3 ppm) and **b** (3 to 6 ppm) spectra collected from obese diabetes group (DG) and normal diet group (NG) Sprague Dawley rats before treatment

The representative ^1^H NMR spectra of serum samples from obese diabetic and normal rats taken at wk0 of treatment are shown in Fig. 6a & b. The relative quantities of identified metabolites with their chemical shift values of obese diabetic and normal rats in serum samples are described in Table 3. 3-hydroxybutyrate, glycerol, acetoacetate, α-glucose, β- glucose, creatine, choline, and taurine were found to be the most dominant metabolites in obese diabetic rats, while isoleucine, acetate, valine, 2-oxoglutarate, succinate, allantoin and hippurate were the most dominant metabolites in serum samples of normal diet rats.

**Table 3:** Relative quantitative (mM) of discriminating metabolites of serum in obese diabetic rats (DG) and normal diet rats (NG) before treatment using Chenomx NMR Suite

Metabolites	Chemical shifts	Direction of Changes in DG	DG	NG
Glucose	5.22	UP	97.484 ± 7.8	32.122 ± 2.4
Pyruvate	3.36	-	0.729 ± 0.05	0.684 ± 0.06
Lactate	1.31	UP	16.413 ± 1.4	14.254 ± 1.2
valine	3.6	DOWN	0.819 ± 0.04	2.147 ± 0.16
2-oxoglutarate	2.42	DOWN	1.907 ± 0.2	3.892 ± 0.3
Succinate	2.39	DOWN	0.792 ± 0.08	1.153 ± 0.1
acetate	1.91	DOWN	1.159 ± 0.08	2.114 ± 0.2
Acetoacetate	3.42	UP	20.514 ± 2.6	7.387 ± 0.5
3-hydroxybutyrate	1.19	UP	7.547 ± 0.6	1.427 ± 0.09
Choline	3.51	UP	17.588 ± 1.8	5.864 ± 0.6
Allantoin	5.38	DOWN	0.449 ± 0.03	0.952 ± 0.06
Creatine	3.91	UP	6.473 ± 0.5	2.181 ± 0.1
Isoleucine	0.92	DOWN	1.111 ± 0.07	1.337 ± 0.1
Hippurate	3.96	DOWN	0.516 ± 0.01	1.640 ± 0.13

### Multivariate data analysis of serum samples of obese diabetic and normal rats

**Fig. 7:**
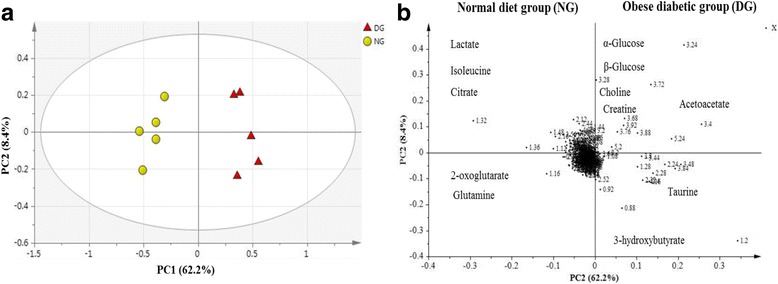
PCA score plot (**a**), loading plot (**b**) obtained using ^1^ H NMR data for serum samples from Sprague-Dawley rats, obese diabetic group (DG) and normal diet group (NG) before treatment

Metabolic differences between the obese diabetic rats and normal rats were unfolded with principal component analysis (PCA) (Fig. 7a). In the present study, R2 = 0.706 and Q2 = 0.500 confirmed the validation of used PCA model. The fitness of the PCA model was also found to be good because the difference between R2 and Q2 was lesser than 0.3 [21]. Distinct separation was observed among the obese diabetic and normal rats (Fig. 7a). The separation was achieved by PC1 (62.2%) and PC2 (8.4%), respectively. The normal rats were located in the negative PC1 region, while obese diabetic rats were clustered distinctly in the positive PC1 region.

The metabolites responsible for the separation between obese diabetic and normal rats are shown in loading pot (Fig. 7b). It was depicted that, 3-hydroxybutyrate, α-glucose, β- glucose, creatine, choline, acetoacetate, and taurine etc., were more prominent in the positive PC1 region (with diabetic obese rat cluster), whereas in the negative PC1 region (with normal rat’s cluster) the most prominent metabolites were lactate, isoleucine, citrate, 2-oxoglutarate and glutamine etc. Based on the results, it can be seen that, metabolic state of the obese diabetic rat group was shifted away from the normal diet group. This shifting reflected the metabolic disturbances and alteration in the metabolites levels resulted from high fat diet (HFD) plus STZ injection. All of these identified metabolites were expected to be involved in different metabolic pathways, including; tricarboxylic acid (TCA) cycle, glucose metabolism, amino acids metabolism, and lipid metabolism.

### Multivariate data analysis of serum NMR data of all groups at week 8 of treatment

**Fig. 8:**
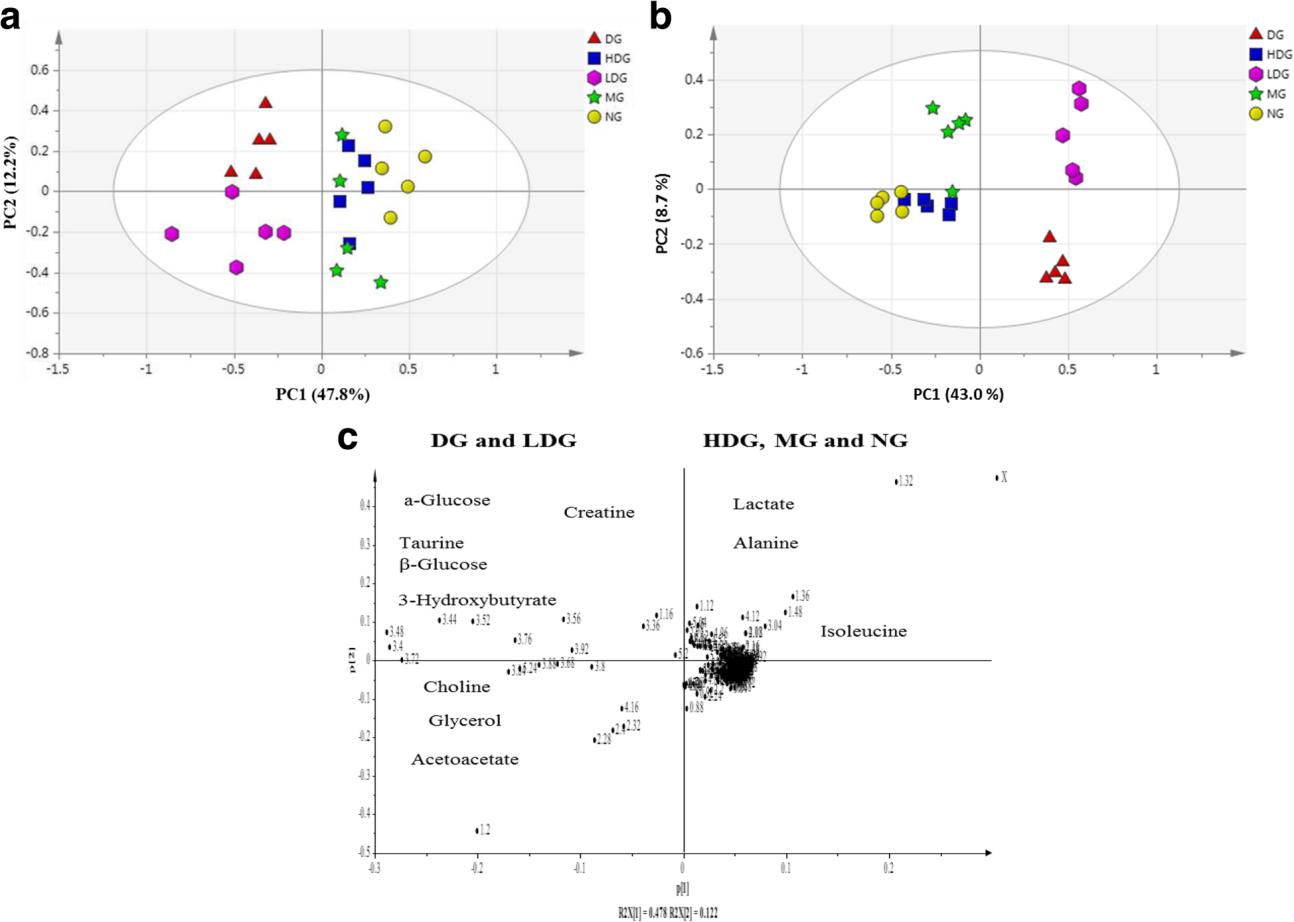
PCA score plot (**a**), OPLS-DA score (**b**) and PCA Loading plot (**c**) obtained using ^1^ H NMR data for serum samples from Sprague-Dawley rats, obese diabetic group (DG), normal diet group (NG), metformin group (MG), low dose group (LDG) or high dose group (HDG) at week8 of treatment

The PCA of serum samples based on NMR data from all the groups i.e., normal diet group (NG), obese diabetic group (DG), metformin group (MG), low dose ML group (LDG) and high dose ML group (HDG) are shown in Fig. 8a. The R2 = 0.736 and Q2 = 0.500 ascertained the validation of used PCA model. Significant metabolic variations among different rat groups after treatment were observed. Good separation was achieved between DG, NG, MG, LDG or HDG after 8 weeks of treatment, and it was depicted that, the selected serum metabolites were significantly changed after treatment with high dose (400 mg/kg BW) of ML extract and metformin (250 mg/kg BW). The separation based on PC1 was 47.8%, while 12.2% separation was by PC2. The DG and LDG were separated from NG, MG and HDG by PC1. The DG and LDG were clustered in -ve PC1region, whereas clustering of NG, MG and HDG was observed in +ve PC1region. Distinct separation in PC1 also indicated a huge difference in the serum metabolome related to the differences among DG, NG, MG, LDG and HDG. However, during the present study a close clustering was observed between NG, MG and HDG, which revealed that treatment with high dose of ML extract produced almost similar effects as that of metformin in bringing the obese diabetic rats toward the state similar to that of normal rats group. Moreover, OPLS-DA was also performed, which revealed more clear separation between NG, MG and HDG (Fig. 8b) with 43.0% separation based on PC1, while 8.7% separation by PC2. Clear separation was observed between obese diabetic rats treated with high dose of ML extract from that of metformin treated at week8, but still not distinctly separated from normal group.

The loading plot (Fig. 8c) describes the metabolites responsible for the separation of DG and LDG from those of NG, MG and HDG. The most dominant metabolites at week8 of treatment in DG and LDG were α-glucose, β-glucose, glycerol, choline, taurine, creatine, 3-hydroxybutyrate, lipoprotein (LDL/VLDL) and acetoacetate. On the other hand, the most dominant metabolites with ML extract and metformin treated and normal group included; lactate, alanine and isoleucine.

Table 4 shows the metabolites which were significantly regulated after treatment with high dose of ML extract comparative to those of untreated obese diabetic groups. A total of 17 metabolite features with VIP values >0.8 were selected. Glucose, acetoacetate, 3-hydroxybutarate (3-HB), choline and creatine were decreased, whereas lactate, formate, 2-oxoglutarate, succinate, hippurate, leucine, isoleucine and alanine were significantly (*p* < 0.05) increased in HDG of ML leaf extract treatment when compared with DG at week8 of treatment, while there were no significant (*p* > 0.05) changes in pyruvate, acetate, allantoin and glutamine levels. Moreover, it was also observed that, the changes in all serum metabolome except lactate were not significant (*p* > 0.05) between MG and HDG at week8 of treatment.

**Table 4:** Relative quantitative (mM) of significant discriminating metabolites of serum using Chenomx NMR Suite at week8 of treatment

Metabolites	Chemical shifts	VIP value	Changes in HDG	DG	NG	MG	LDG	HDG
Glucose	5.22 3.89	1.75 1.63	DOWN	386.2 ± 11.8^a^	150.4 ± 13.6^d^	175.8 ± 4.3^c^	260.5 ± 9.7^b^	171.3 ± 2.8^cd^
Pyruvate	3.37	1.34	-	0.91 ± 0.15^b^	1.77 ± 0.17^a^	1.50 ± 0.16^ab^	1.15 ± 0.13^ab^	1.46 ± 0.06^ab^
Lactate	1.32	0.90	UP	18.55 ± 6.45^c^	51.21 ± 2.63^a^	44.72 ± 5.33^a^	25.76 ± 3.24^bc^	30.07 ± 1.61^b^
Formate	8.44	0.47	UP	0.27 ± 0.02^d^	0.79 ± 0.13^a^	0.54 ± 0.05^bc^	0.35 ± 0.03^cd^	0.61 ± 0.05^ab^
2-oxoglutarate	2.42	2.26	UP	0.98 ± 0.06^c^	3.96 ± 1.27^a^	2.63 ± 0.14^ab^	1.67 ± 0.13^bc^	2.78 ± 0.15^ab^
Succinate	2.40	2.54	UP	0.62 ± 0.10^b^	1.48 ± 0.35^a^	1.21 ± 0.14^a^	0.97 ± 0.23^ab^	1.19 ± 0.06^a^
Acetate	1.91	0.61	-	1.13 ± 0.05^a^	1.56 ± 0.10^a^	1.27 ± 0.15^a^	1.16 ± 0.14^a^	1.32 ± 0.12^a^
Acetoacetate	3.42	3.40	DOWN	51.47 ± 5.0^a^	27.56 ± 1.02^b^	29.24 ± 4.49^b^	42.0 ± 5.11^a^	25.98 ± 1.65^b^
3-HB	1.2	5.50	DOWN	43.55 ± 6.76^a^	6.53 ± 0.68^b^	11.03 ± 0.83^b^	14.49 ± 1.19^b^	7.87 ± 0. 53^b^
Choline	3.52	2.34	DOWN	40.30 ± 2.71^a^	18.96 ± 1.22 ^c^	24.90 ± 0.35^bc^	30.92 ± 4.09^b^	19.66 ± 1.55^c^
allantoin	5.38	0.98	-	0.91 ± 0.05^b^	1.54 ± 0.16^a^	1.18 ± 0.13^ab^	0.99 ± 0.15^ab^	1.15 ± 0.10^ab^
Creatine	3.91	1.43	DOWN	12.90 ± 2.20^a^	9.34 ± 0.59^b^	11.55 ± 1.20^ab^	12.67 ± 0.30^a^	8.71 ± 0.84^b^
glutamine	3.77	2.12	-	33.54 ± 6.23^a^	32.067 ± 1.71^a^	27.60 ± 3.09^a^	33.86 ± 1.52^a^	28.30 ± 1.70^a^
Leucine	0.95	0.87	UP	0.84 ± 0.07^b^	1.45 ± 0.17^a^	1.41 ± 0.07^a^	0.87 ± 0.05^b^	1.38 ± 0.09 ^a^
isoleucine	1.0	0.57	UP	1.35 ± 0.13^b^	2.50 ± 0.25^a^	2.32 ± 0.18^a^	1.43 ± 0.20^b^	2.26 ± 0.16^a^
hippurate	3.95	0.63	UP	2.36 ± 0.06^c^	8.57 ± 0.54^a^	4.54 ± 0.36^b^	3.12 ± 0.41^c^	4.90 ± 0.26^b^

Significance difference (*p* < 0.05) between the different groups is shown by different letters

Interestingly, the biochemical changes of the obese diabetic rats were gradually restored to normal levels after administration of MG and HDG, implying that ML exhibits significant efficacy regarding the improvement of metabolism disorders in obese diabetic rats.

### Metabolites analysis of serum NMR data of ML extract and Metformin at different periods of treatment

**Fig. 9:**
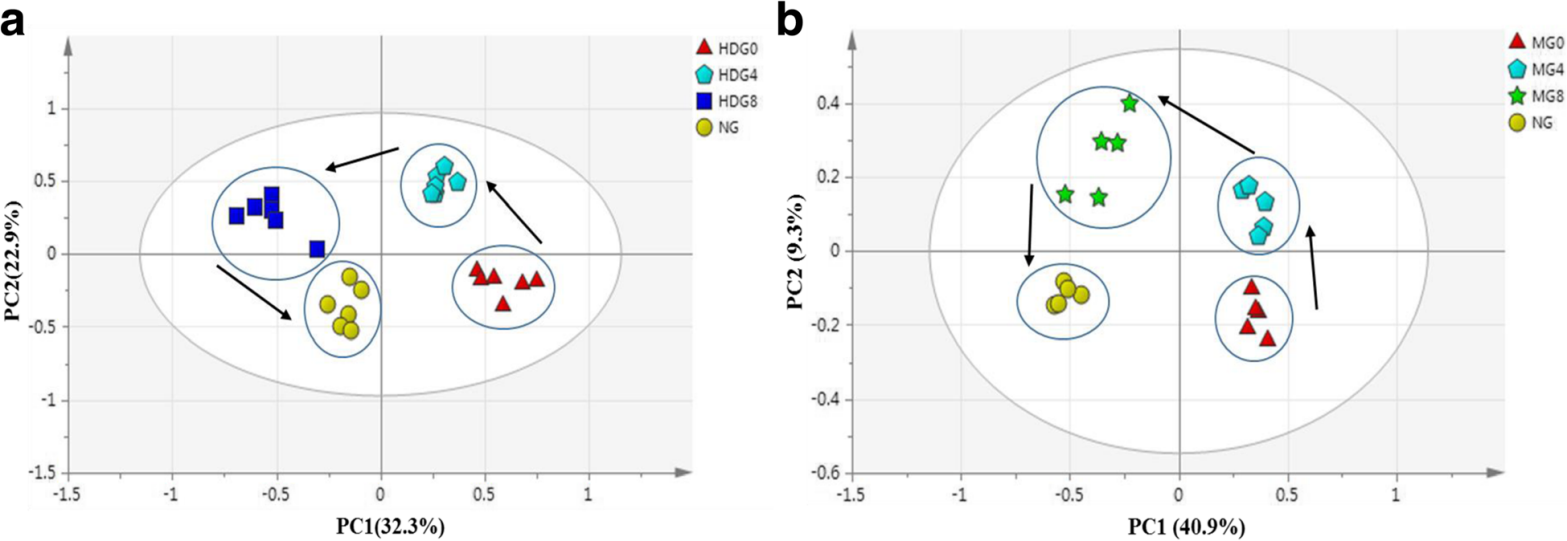
OPLS-DA score plot of (**a**) ML and (**b**) metformin obtained using ^1^ H NMR data for serum samples from Sprague-Dawley rats, normal diet group (NG), week0 (HDG0 and MG0), week4 (HDG4 and MG4) and week8 (HDG8 and MG8) of treatment

An OPLS-DA model was employed for the binned NMR spectra of the serum samples collected from obese diabetic rats at week0 (HDG0, MG0), week4 of treatment (HDG4, MG4) and at week8 of treatment (HDG8, MG8), respectively comparative to the normal group (NG) rats (Fig. 9a & b). After auto-fitting, the OPLS-DA analysis gives good statistical performances and score plots of ML extract and metformin treated groups (R2Y = 0.883, Q2Y = 0.835 and R2Y = 0.879, Q2Y = 0.749, respectively) for the first two components (PC1 and PC2) with clustering based on treatment time.

In case of ML extract treated group, the PC1 presented 32.3%, whereas the PC2 presented 22.9% of total separation. However, for metformin treated groups, 40.9% and 9.3% separations were due to PC1 and PC2, respectively. The trajectories of metabolite changes among week0, week4 and week8 of treatments with ML extract and metformin are shown in Fig. 9a & b, respectively.

The week0 cluster migrations were observed during the treatment with ML extract (HDG0) from +ve PC1 to +ve PC2 (week4) after having a treatment of 4 weeks (Fig. 9a). It was observed that after treatment with ML extract for 8 weeks, the trajectories of the cluster migrations were shifted towards the positive region of the PC2 (HDG8). The changing trend in the metabolites pattern is denoted by arrows. From these clear migratory characteristics, it was found that, the long term treatment with ML extract restored the metabolic state of the obese diabetic rats towards the normal conditions. The cluster migrations in case of metformin treatment were also similar to those resulting from the treatment of ML extract (Fig. 9b).

**Fig. 10:**
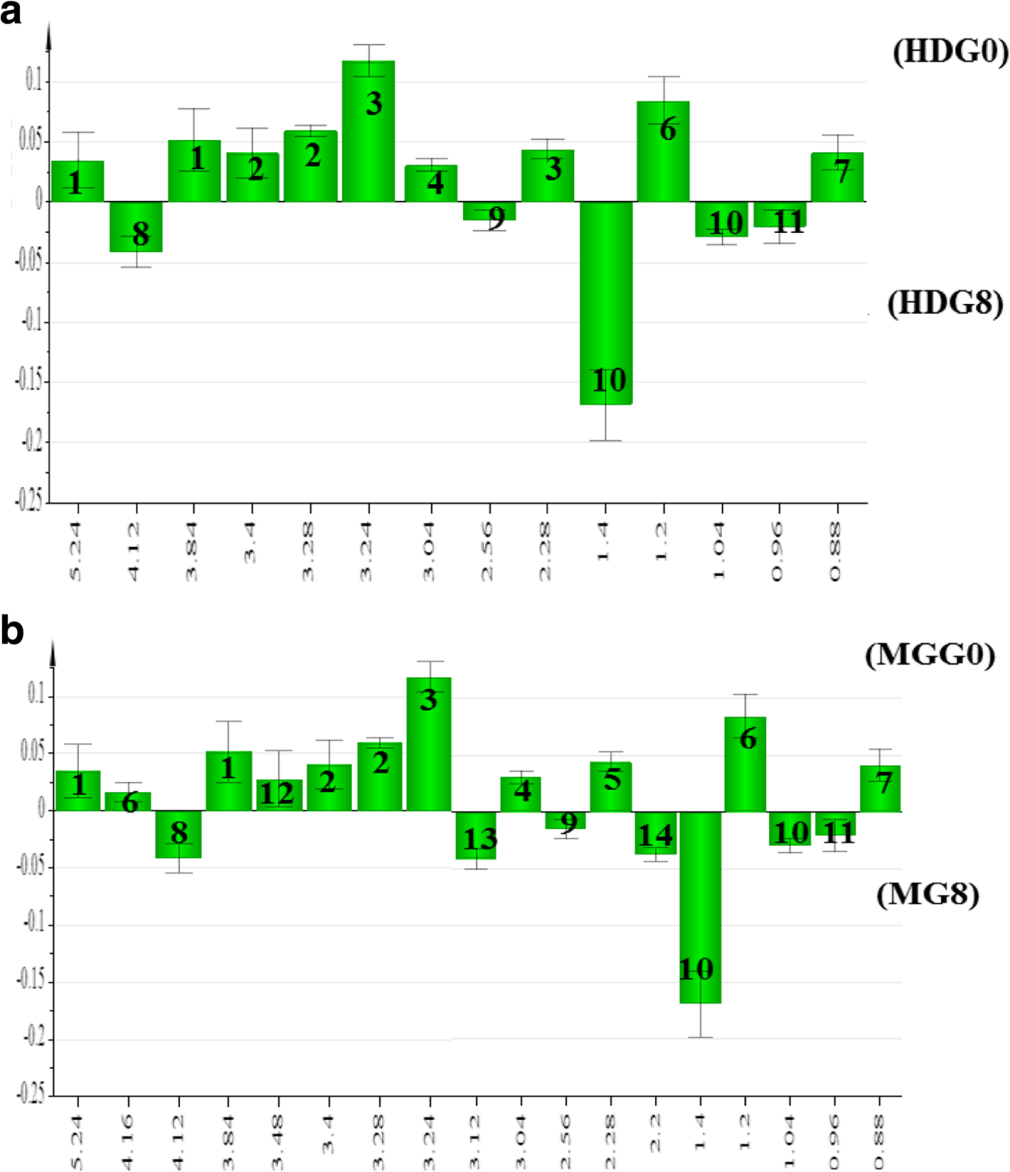
OPLS-DA loading column plot of (**a**) ML and (**b**) metformin obtained using ^1^ H NMR data for serum samples from Sprague-Dawley rats, Week0 (HDG0), and Week8 (HDFG8). For ML: 1; α-glucose, 2; taurine, 3; choline, 4; creatine, 5; acetoacetate, 6; 3-hydroxybutyrate, 7; 2-hydroxybutyrate, 8; lactate, 9; citrate, 10; Isoleucine,11; leucine. For metformin; 1; α-glucose, 2; taurine, 3; choline, 4; creatine, 5; acetoacetate, 6; 3-hydroxybutyrate, 7; 2-hydroxybutyrate, 8; lactate, 9; citrate, 10; Isoleucine,11; leucine, 12; β-glucose, 13; creatinine, 14; valine

The column plots (Fig. 10a & b) describe the metabolites responsible for the separation before and after treatment with ML extract and metformin, respectively. Before treatment with ML extract, α-glucose, choline, taurine, creatine, acetoacetate, 3-hyroxybutyrate and 2-hyroxybutyrate were more dominant biomarkers; however, after treatment the most dominant metabolites include; lactate, isoleucine, citrate and leucine (Fig. 10a).

On the other hand, in case of metformin treatment, α-glucose, 3-hyroxybutyrate, β-glucose, creatine, choline, acetoacetate, taurine and 2-hyroxybutyrate were dominant biomarkers before treatment, while lactate, creatinine, citrate, valine, isoleucine and leucine were the most prominent metabolites after treatment with metformin (Fig. 10b). Conclusively, these metabolites may be considered as potential biomarkers for differentiation among diabetic and healthy rats. The results thus ascertained the recovery trend of ML extract treated rats toward the normal rats.

## Discussion

Medicinal plants are rich source of polyphenols and thus, could present a cheaper, safer and more effective option for managing T2DM than the synthetic antidiabetic agents. Polyphenols exhibit many pharmacological activities including antidiabetic properties [22]. Our previous in vitro studies ascertained that, the *Melicope lunu-ankenda* (ML) leaf extract is enriched with numerous polyphenols including isorhamnetin, skimmianine, scopoletin and melicarpinone [17]. During the present study, polyphenols rich ethanolic extract of ML was revealed to exhibit potent anti-diabetic activities in vivo study.

The combination of HFD and low dose of STZ is a well-accepted approach for the induction of T2DM in animals, which is characterized by hyperglycemia, hyperlipidemia, and hyperinsulinemia [3]. HFD usually results in obesity leading to insulin resistance, whereas low dose of STZ causes moderate destruction of insulin secretion [23].

In the present study, obesity was build up in animals before induction of diabetes. The study showed noticeable development in body weights of rats in response to HFD diet compared to that of normal diet. An increase in body weight of rats fed with HFD due to excessive fat build up can impair rat’s health [24]. However, the HFD/STZ induced diabetic rats group (DG) showed drastic weight loss after STZ induction when compared with that of normal diet group (NG). This may be due to dehydration and catabolism of the fat and protein. However, the obese diabetic rats treated with ML extract (200 and 400 mg/kg BW) and metformin (250 mg/kg BW) significantly prevented the body weight loss (Fig. 3) and restored up to the level of healthy rats, which might be due to the regulation of the hyperglycaemic state. The stabilized blood glucose in the diabetic rats treaded with ML extract and metformin could also results in the steady body weights. At week0, a significant (*p* < 0.05) increase in blood glucose level was observed in obese diabetic rats when compared with that of healthy rats before treatment. However, there was no significant (*p* > 0.05) difference in case of insulin levels. The results were in agreement with that reported by [24], who reported that HFD/STZ rat models with raised blood glucose levels coexist with either the same, lower or higher levels of insulin when compared with that of control. It was also observed in present study, that the obese diabetic rats consumed more water comparative to the healthy ones before treatment. However, obese diabetic rats after ML extract treatment consumed comparatively lesser water, which may be due to enhanced glycemic status. These observations were in agreement with those reported by [25].

At the end of the treatment with 400 mg/kg BW of ML extracts and 250 mg/kg BW of metformin, significant differences were observed in the insulin levels compared to those of untreated obese diabetic rats, and the level was closer to those of healthy rats. All the obese diabetic rats exhibited significantly higher insulin resistance levels compared to the healthy ones before treatment. At the completion of treatment period, the obese diabetic rats treated with ML leaf extract and metformin showed a significant decrease in the insulin resistance level compared to that of untreated obese diabetic group. The insulin sensitivity of obese diabetic rats was also enhanced after being treated with ML leaf extract, hence demonstrated that the ML Leaf extract exhibited significant insulin sensitization activity leading to enhanced glucose homeostasis. This was probably due to improved pancreatic ß-cell action as evident from enhanced serum insulin level. The results of the current study demonstrated that, ML extract may be effective in enhancing the condition of β-cells in the Langerhans islet, as it may have improved insulin secretion. In addition, an opposite correlation between the insulin resistance and insulin sensitivity in a dose dependent manner of ML extract treated rats was observed, which could propose that, the extract may be responsible for the sensitization of insulin binding action for cellular glucose uptake. Similar type of observations have also been reported in a previous study, where green tea treated diabetic rats showed improved insulin sensitivity by enhancing the insulin affinity to bind with adipocytes [26].

Diabetic hyperglycaemia is known to be associated with alterations in blood lipid profile. These changes in blood lipid profile occur due to irregularities in the lipid metabolism, and result in the progression of coronary heart disease (CHD) in diabetic patients [27]. Under usual metabolic conditions, insulin induces lipoprotein lipase, which is the enzyme that hydrolyses triglycerides to produce glycerol and fatty acids. The fatty acids then get absorb by the body tissues, where they are either oxidized for energy or re-esterified for storage. However, lack in insulin results in inhibition of this enzyme thus leading to increase TG level [11].

The present study also demonstrated irregularities in the lipid metabolism, significant elevation in serum TC, TG, LDL, while reduction in serum HDL levels was observed in the obese diabetic rats before treatment. However, treatment with ML extract significantly suppressed the TC, TG and LDL in obese diabetic rats, whereas HDL levels were elevated in dose dependent manner. Results of the study were in agreement with those of previous study, where hamsters rats fed with high-fat diet following treatment of *Schisandrachinensis* fruit (SF) extract showed significant reduction in their serum levels of TC, TG, LDL and enhancement in HDL level [28]. Similar results have also been reported in another study, where diabetic rats treated with O-prenylated flavonoid (3,5,40-trihydroxy-8,30-dimethoxy-7-(3-methylbut-2-enoxy)flavone;OPF) isolated from *Melicope lunu-ankenda* leaves showed decrease in serum TC and TG levels, with increased levels of HDL [29]. The observed reduction in the elevated serum TC, TG, and LDL levels and augmentation in HDL level of obese diabetic rats fed with ML extract might be related to the enhanced insulin secretion by the pancreatic β-cells, which is an indicator of lipid metabolism and utilization. The ability of ML extract to decrease TC, TG, and LDL levels to the normal levels and to increase HDL level in obese diabetic rats reduces the risk of cardiovascular troubles associated with diabetes mellitus. Therefore, ML leaf extract also prevented diabetic linked complications in diabetic rats by regulating their lipid levels.

The liver enzymes are well recognized as marker enzymes of liver damage. The hepatoprotective effects of the ML leaf extract treated obese diabetic rats were revealed from the significant reduction in the levels of liver enzymes i.e., AST, ALT and ALP in obese diabetic rats (after 8 weeks) in a dose dependent manner. Therefore, the ML leaf extract treatment restored the liver functions in obese diabetic rats to the normal levels. Similar type of effect has also been reported by [30].

In the current study, before treatment, the obese diabetic rats showed elevated urea and creatinine levels, which were then reverted back to the levels of healthy rats upon treatment of ML leaf extract. The effect was similar to that observed in the obese diabetic rats treated with metformin. These findings were in agreement with previous study conducted by [31]. The ML extract therefore prevented the kidney damage which could be possible in diabetic rats [31]. The levels of these metabolites rise in blood during renal dysfunction or renal diseases associated with untreated diabetes [32]. Therefore, serum urea and creatinine may be considered as important markers of renal damage.

As expected, the diabetic rats showed higher liver weights. The higher liver weights of obese diabetic rats might be due to fibrosis or abnormal glycosylation referred to as hepatic steatosis. However, the liver weights of ML extract treated rats were significantly decreased as compared with those of untreated obese diabetic rats, to the levels almost equivalent to the healthy rats. In a previous study, diabetic rats treated with OPF isolated from *Melicope lunu-ankenda* leaves also restored the liver weights to the normal levels [29]. In addition, the treatment of obese diabetic rats with ML leaf extract decreased the weight of kidney (Table 2) after 8 weeks of treatment. This observation was in accordance with previous study [33].

The ^1^HNMR-based serum metabolomics studies clearly differentiated the understudy rat groups before and after treatment with ML extract, and also identified the potential biomarkers along with major alteration in different metabolic pathways. The results revealed the successfulness of the artificial type 2 diabetes model using rats. It was observed that, the pathological conditions were improved, and the metabolite state was reversed back toward the normal levels with the administration of ML extract. Our findings were in agreement with those reported by [32]. Compared with ML extract treated obese diabetic rats and healthy rats, the obese diabetic rats exhibited higher levels of glycerol, acetoacetate and 3-hydroxybutyrate (3-HB) at week8 (Fig. 10 and Table 4), demonstrating an impaired storage of circulating fatty acids in adipose tissue, inhibited hepatic fatty acid esterification and disturbed ketone body’s metabolism [34]. It was revealed that, the obese diabetic rats treated with ML extract significantly up-regulated the levels of 2-oxoglurarate, succinate, and citrate at week8 (Table 4). The maintenance of the glucose homeostasis also requires fully-functioned TCA cycle with ability to produce gluconeogenesis precursors and facilitate glucose oxidation [35].

**Fig. 11:**
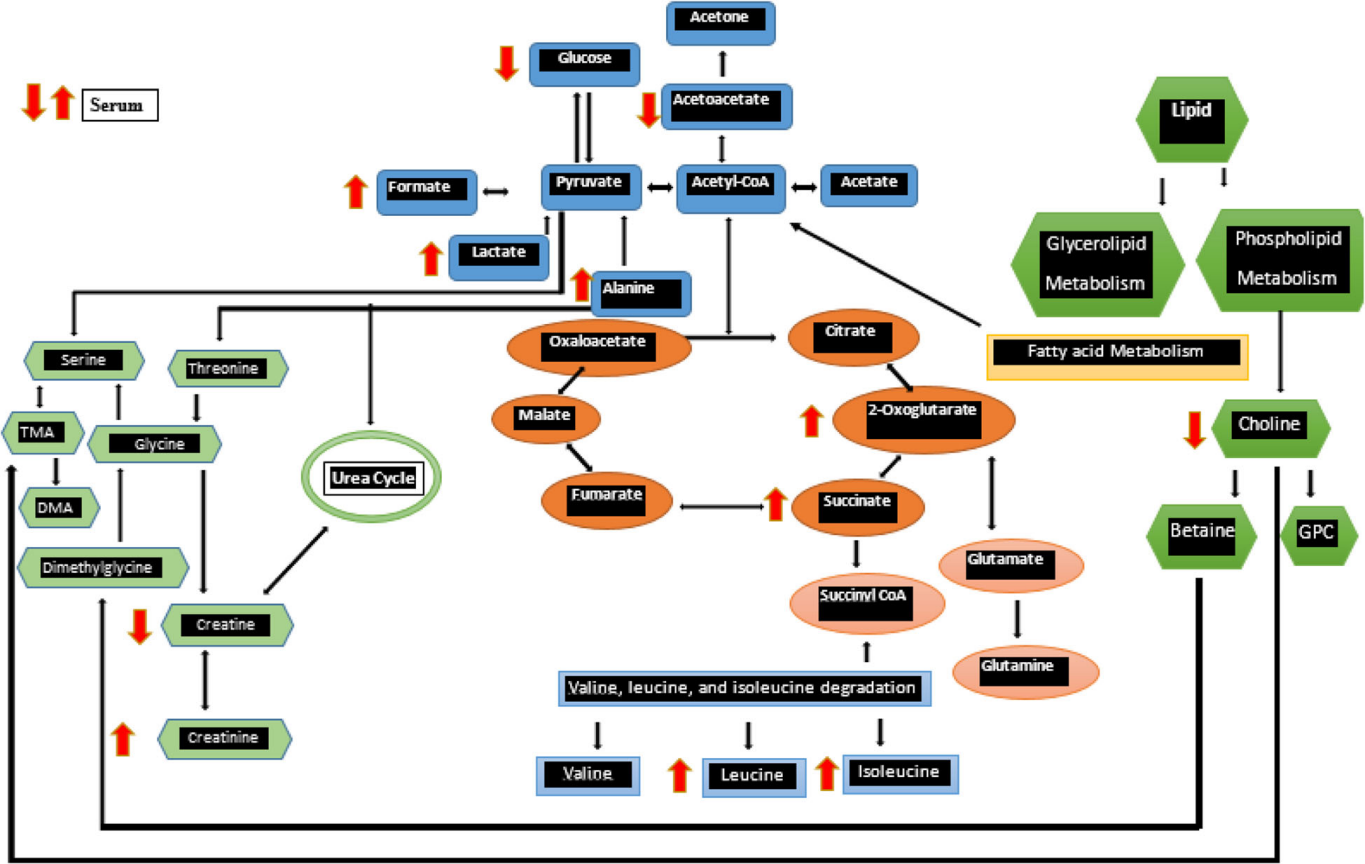
Metabolism pathways describing regulation of potential urine and serum biomarkers upon ML extract treatment

A visible decrease in amino acids (leucine, isoleucine) levels was observed in diabetic rats. This decrease reflects an increase in protein metabolism resulted from up-regulated gluconeogenesis and ketone body’s metabolism pertaining to diabetes mellitus [32]. The treatment of obese diabetic rats with ML extract significantly up-regulated the levels of amino acids (leucine, isoleucine) at week8 of treatment (Table 4). In general, the ML extract treatment partially recovered the metabolism disorders of obese diabetic rats and exerted good antidiabetic effects. The observed alterations in different metabolic pathways are described in Fig. 11.

## Conclusions

In summary, the present study ascertained the pharmacological significance of ethanolic ML leaf extract for the treatment of diabetes mellitus. Treatment of obese diabetic rats with ML leaf extract significantly increased insulin sensitivity, with concomitant decrease in the level of insulin resistance. The diets supplemented with ML leaf extract exhibited potent antidiabetic activity in T2DM rats, by improving their lipids profile and insulin sensitivity. The high-throughput NMR based metabolomics approach successfully identified the potential serum biomarkers for T2DM and explored the underlying changes in the metabolic pathways during the treatment of obese diabetic rats with ML extract. Conclusively, the ML leaf extract could be a useful and affordable alternative option for the management of T2DM.

## Abbreviations

ALP: Alkaline Phosphatase
ALT: Alanine Aminotransferase
AST: Aaspartate Aminotransferase
BW: Body Weight
CMC: Carboxymethyl Cellulose
CPMG: Carr-Purcell Meiboom-Gill
ELISA: Enzyme-Linked Immune Absorbent Assay
HDG: High Dose Group
HDL: High Density Lipoprotein
HFD: High Fat Diet
IACUC: Institutional Animal Care and Use Committee
LDG: Low Dose Group
LDL: Low Density Lipoprotein
MG: Metformin Group
ML: *Melicope lunu-ankenda*
ND: Normal Diet
NG: Normal Group
NMR: Nuclear Magnetic Resonance
OPLS-DA: Orthogonal Principal Least Squire-Discriminatory Analysis
PCA: Principal Component Analysis
ROS: Reactive oxygen species
STZ: Streptozotocin
T2DM: Type 2 diabetes mellitus
TC: Total Cholesterol
TG: Triglyceride
TSP: Trimethylsilanepropionic Acid Sodium Salt
UPM: University Putra Malaysia
